# Dramatic, significant metabolic response to a one‐time pembrolizumab treatment following a relapse of pre‐existing organizing pneumonia in a patient with advanced non‐small cell lung cancer: A case report

**DOI:** 10.1111/1759-7714.14185

**Published:** 2021-10-06

**Authors:** Motoyasu Kato, Mikiko Mori, Keita Miura, Tetsuhiko Asao, Hiroaki Motomura, Koichi Nishino, Ryo Ko, Ryo Koyama, Takuo Hayashi, Kazuhisa Takahashi

**Affiliations:** ^1^ Department of Respiratory Medicine Juntendo University Graduate School of Medicine Tokyo Japan; ^2^ Department of Human Pathology Juntendo University Graduate School of Medicine Tokyo Japan

**Keywords:** drug‐induced interstitial pneumonia, immune checkpoint inhibitors, immune‐related adverse event, non‐small cell lung carcinoma, pembrolizumab

## Abstract

Immune checkpoint inhibitors can often trigger immune‐related adverse events (irAEs), such as relapse of pre‐existing interstitial pneumonia. Here, we report the case of an 88‐year‐Japanese man diagnosed with advanced lung adenocarcinoma with a high tumor proportion score of programmed death‐ligand 1. Six years earlier, he had developed organizing pneumonia (OP), a subtype of interstitial pneumonia, that was treated with steroid pulse therapy maintained with prolonged prednisolone administration. We initiated pembrolizumab as the first‐line treatment. One month after the first pembrolizumab administration, high resolution computed tomography (HRCT) of the chest demonstrated ground‐glass opacities and consolidations. We suspected pembrolizumab‐induced OP relapse, an irAE. His oxygenation was normal; therefore, we discontinued pembrolizumab without additional treatment for OP relapse. Four months after OP relapse, HRCT showed no new findings. After significant amelioration of OP, although the size of the tumor shadow remained the same on HRCT, positron emission tomography‐computed tomography demonstrated the disappearance of the standardized uptake value of the primary tumor, mediastinal lymph nodes, and pleural nodules. In conclusion, this is the first report of a dramatic, significant metabolic response after a single pembrolizumab treatment despite the relapse of pre‐existing OP in a patient with advanced lung adenocarcinoma.

## Graphical abstract

High resolution computed tomography findings of the chest at (a) organizing pneumonia (OP) occurrence, (b) after OP amelioration, (c) diagnosis of lung cancer, (d) relapse of OP induced by pembrolizumab treatment, and (e) after amelioration of OP relapse.

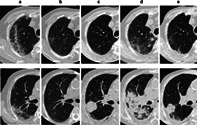

## INTRODUCTION

Patients with pre‐existing interstitial pneumonia may easily be predisposed to immune checkpoint inhibitor (ICI)‐induced interstitial lung disease or ICI‐related progression/relapse of the disease compared with patients without pre‐existing interstitial pneumonia.^1^ Although survival time is reportedly longer in patients who develop immune‐related adverse events (irAEs) during ICI treatment than in those who do not^1^, ^2^, ^3^ in the real world, the association between irAE development, particularly ICI‐induced interstitial pneumonia, and the efficacy of ICI in reducing the tumor size is yet to be completely elucidated. Here, we report the first case of a patient with advanced non‐small cell lung cancer (NSCLC) who developed ICI‐induced relapse of pre‐existing organizing pneumonia (OP). The patient improved without additional treatment for OP and was cured of advanced NSCLC by a single pembrolizumab administration.

## CASE REPORT

**FIGURE 1:**
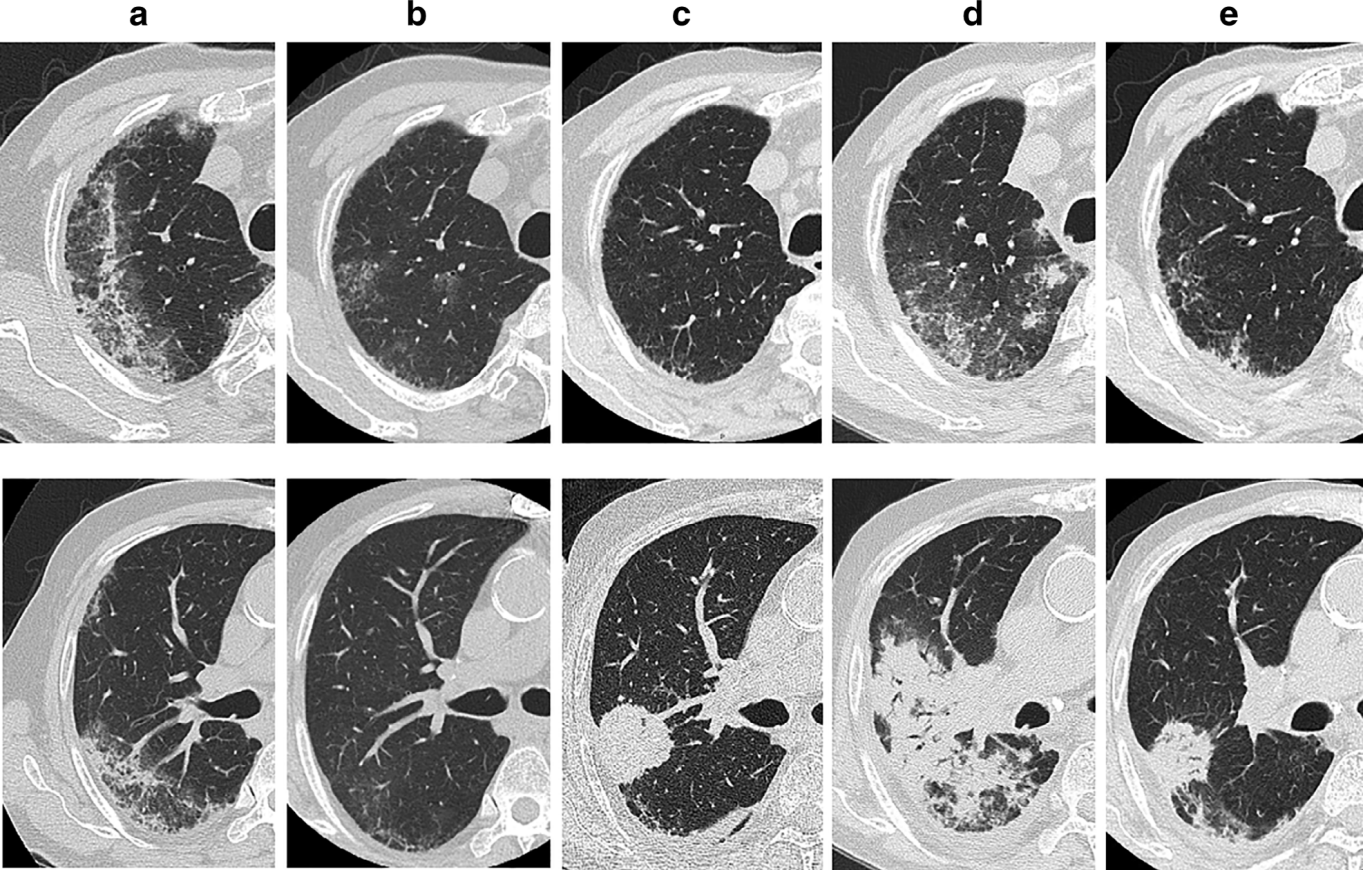
High resolution computed tomography (CT) of the chest at (a) organizing pneumonia (OP) occurrence, (b) after OP amelioration, (c) diagnosis of lung cancer, (d) relapse of OP, and (e) after amelioration of OP relapse

**FIGURE 2:**
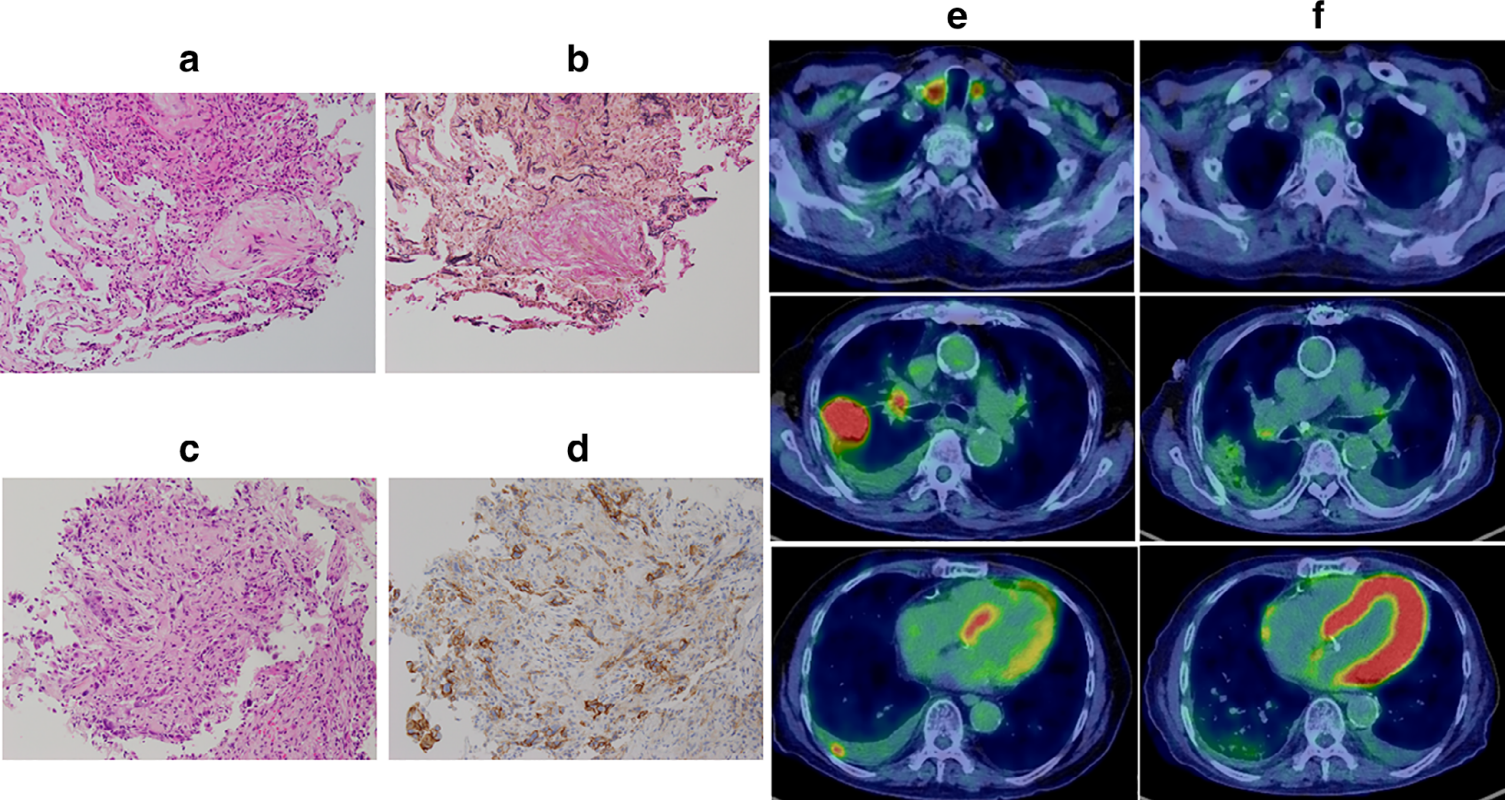
Pathology and positron emission tomography‐computed tomography (PET‐CT) findings. Pathology findings of the lung at OP occurrence with hematoxylin and eosin (HE) (a) and Elastica van Gieson staining (b), and at the diagnosis of lung cancer with HE (c) and programmed cell death 1 staining (d). PET‐CT findings at the diagnosis of lung cancer (e) and 18 months after initiation of pembrolizumab (f)

An 82‐year‐old Japanese man who had been diagnosed with OP 6 years prior was diagnosed with lung adenocarcinoma based on a new nonsegmental bilateral consolidation and ground‐glass opacity (GGO) on chest high‐resolution computed tomography (HRCT) (Figure 1a) and infiltration of inflammatory cells. Transbronchial lung biopsy revealed that the alveoli were occupied by fibroblast nodules and immature connective tissues (Figure 2a,b). Corticosteroids were administered, and HRCT findings were ameliorated thereafter (Figure 1b). At 88 years of age, the patient was diagnosed with adenocarcinoma (Figure 2c), with a wild‐type epidermal growth factor receptor mutation. Transbronchial biopsy (Figure 2d) revealed a tumor proportion score (TPS) of 70%. The tumor was classified as stage IVA (T4N3M1a) based on right lung findings, supraclavicular lymph node swelling on HRCT (Figure 1c), and malignant pleural effusion. Positron emission tomography‐computed tomography (PET‐CT) showed a high standardized uptake value (SUV_max_ > 10), with a primary tumor mass shadow, mediastinal lymph nodes, and pleural nodules (Figure 2d). We initiated ICI monotherapy with pembrolizumab as the first line of chemotherapy based on the TPS of 70% and patient's old age. One month after the first pembrolizumab administration, bilateral consolidations and GGO appeared on HRCT. We considered OP relapse due to pembrolizumab treatment and interrupted the treatment but continued with administration of the corticosteroid. Four months after OP relapse, HRCT findings worsened (Figure 1d). However, oxygenation in the patient remained stable; thus, we did not administer additional treatment for OP. Six months after OP relapse, improvements were noted on HRCT following the continuation of corticosteroid therapy alone (Figure 1e). Eighteen months after the initiation of pembrolizumab, PET‐CT demonstrated a decrease in SUV_max_ in the primary tumor and all metastatic lesions (Figure 2e). Therefore, we considered this a dramatic, significant metabolic response with only a one‐time pembrolizumab administration.

## DISCUSSION

In our case, HRCT findings on presentation were similar to the previous OP findings; therefore, based on the radiological findings and past history, we considered an ICI treatment‐induced OP relapse. According to The Common Terminology Criteria for Adverse Events, the pneumonitis grade of the patient was 1; therefore, we did not increase the corticosteroid dose.^4^

The association between ICIs and corticosteroids remains unclear. Many clinical trials have indicated the insufficiency of steroids for ICI treatment‐based tumor regression. Further, it has been reported that prognosis is significantly poorer in patients with NSCLC who receive over 10 mg of prednisone than in those who receive less for palliative indications.^5^ Moreover, ICI treatment has been reported to exacerbate autoimmune disease.^6^ We usually manage worsening autoimmune diseases in such cases with corticosteroids. Quite interestingly, IrAE development may be associated with a good response to ICIs; thus, corticosteroids for irAEs may inhibit the effect of ICIs on tumor progression.

In the present case, a one‐time pembrolizumab treatment induced a significant metabolic response. Our report suggests that a single ICI treatment may effectively inhibit tumor cell proliferation. Moreover, we did not increase the dose of corticosteroids, which may have improved the effect of pembrolizumab on the tumor.

This patient received corticosteroid treatment because OP relapsed. When OP relapsed, we first initiated prednisolone treatment at a dose of 0.5 mg/kg (30 mg/day), followed by steroid pulse therapy. Then, we gradually reduced the corticosteroid dose to 3 mg/day by 1–5 mg/day every 2 weeks for 10 weeks. When we decreased the corticosteroid dose by 3 mg, his chest radiological findings became slightly worse. Hence, the 3 mg/day prednisolone dose was used as maintenance therapy for over 5 years in this patient. Although OP relapsed in the patient, we did not increase the prednisolone dose but maintained it at 3 mg/day. Since we did not change the corticosteroid dose from 3 mg/day after the development of adenocarcinoma and OP relapse, we hypothesize that corticosteroid treatment was unrelated to the effect of pembrolizumab and OP relapse.

The frequency of complete response is significantly higher in patients who receive either ICI treatment or ICIs in addition to cytotoxic chemotherapy agents than in patients who receive cytotoxic chemotherapy alone.^7^ Additionally, many reports have shown a complete response to ICI treatment in patients with NSCLC.^8^ These cases were administered ICI treatment multiple times until a complete response was achieved. On the other hand, our patient showed a significant metabolic response despite only a single ICI treatment. This is the first case of NSCLC that achieved a dramatic, significant metabolic response after only a one‐time pembrolizumab treatment.

The efficacy and safety of pembrolizumab monotherapy for elderly patients has been retrospectively investigated in Japan,^9^ and they are reportedly similar to those for individuals of all ages. In this previous study, two patients achieved complete response (4.6%), and three developed interstitial pneumonia (6.3%) of any grade. However, the association between pembrolizumab efficacy and interstitial pneumonia occurrence as an irAE could not be examined because of the small number of patients in the study. A comparison of our patient with the two patients who achieved a complete response in the previous study showed that all patients had good performance status (0–1). Although the number of patients was small, a good performance status may be one of the effective markers for pembrolizumab monotherapy in elderly patients with NSCLC.

In conclusion, we report an extremely important NSCLC case who achieved a significant metabolic response after a one‐time ICI treatment and recovery after the discontinuation of pembrolizumab treatment due to relapse of pre‐existing OP, an irAE.

## CONFLICT OF INTEREST

The authors declare that they have no competing interests.

## References

[1] Sugano T , Seike M , Saito Y , Kashiwada T , Terasaki Y , Takano N , et al. Immune checkpoint inhibitor‐associated interstitial lung diseases correlate with better prognosis in patients with advanced non‐small‐cell lung cancer. Thorac Cancer. 2020;11:1052–60. 32096610 10.1111/1759-7714.13364 PMC7113045

[2] Haratani K , Hayashi H , Chiba Y , Kudo K , Yonesaka K , Kato R , et al. Association of immune‐related adverse events with nivolumab efficacy in non‐small cell lung cancer. JAMA Oncol. 2018;4:374–8. 28975219 10.1001/jamaoncol.2017.2925 PMC6583041

[3] Teraoka S , Fujimoto D , Morimoto T , Kawachi H , Ito M , Sato Y , et al. Early immune‐related adverse events and association with outcome in advanced non‐small cell lung cancer patients treated with Nivolumab: a prospective cohort study. J Thorac Oncol. 2017;12:1798–805. 28939128 10.1016/j.jtho.2017.08.022

[4] Puzanov I , Diab A , Abdallah K , Bingham C , Brogdon C , Dadu R , et al. Managing toxicities associated with immune checkpoint inhibitors: consensus recommendations from the Society for Immunotherapy of Cancer (SITC) Toxicity Management Working Group. J Immunother Cancer. 2017;5:95. 29162153 10.1186/s40425-017-0300-z PMC5697162

[5] Ricciuti B , Dahlberg SE , Adeni A , Sholl LM , Nishino M , Awad MM . Immune checkpoint inhibitor outcomes for patients with non‐small‐cell lung cancer receiving baseline corticosteroids for palliative versus nonpalliative indications. J Clin Oncol. 2019;3:1927–34. 10.1200/JCO.19.00189 31206316

[6] Abdel‐Wahab N , Shah M , Lopez‐Olivo MA , Suarez‐Almazor ME . Use of immune checkpoint inhibitors in the treatment of patients with cancer and preexisting autoimmune disease: a systematic review. Ann Intern Med. 2018;168:121–30. 29297009 10.7326/M17-2073

[7] Gandhi L , Rodríguez‐Abreu D , Gadgeel S , Esteban E , Felip E , de Angelis F , et al. Pembrolizumab plus chemotherapy in metastatic non‐small‐cell lung cancer. N Engl J Med. 2018;378:2078–92. 29658856 10.1056/NEJMoa1801005

[8] Takeuchi E , Okamoto Y , Takahashi N , Morizumi S , Toyoda Y , Kuroda N , et al. Complete response of squamous cell carcinoma of the lung following treatment with pembrolizumab in an elderly patient: a case report. Thorac Cancer. 2020;2:259–63. 10.1111/1759-7714.13733 PMC7812064 33174378

[9] Imai H , Wasamoto S , Yamaguchi O , Suzuki K , Sugiyama T , Uchino J , et al. Efficacy and safety of first‐line pembrolizumab monotherapy in elderly patients (aged ≥ 75 years) with non‐small cell lung cancer. J Cancer Res Clin Oncol. 2020;146:457–66. 31853661 10.1007/s00432-019-03072-1 PMC11804284

